# Nano-silver-incorporated biomimetic polydopamine coating on a thermoplastic polyurethane porous nanocomposite as an efficient antibacterial wound dressing

**DOI:** 10.1186/s12951-018-0416-4

**Published:** 2018-11-12

**Authors:** Menglong Liu, Tengfei Liu, Xiwei Chen, Jiacai Yang, Jun Deng, Weifeng He, Xiaorong Zhang, Qiang Lei, Xiaohong Hu, Gaoxing Luo, Jun Wu

**Affiliations:** 1 Institute of Burn Research, State Key Laboratory of Trauma, Burn and Combined Injury, Southwest Hospital, Third Military Medical University (Army Medical University), Chongqing, 400038 China; 2 Department of Urology, Second Affiliated Hospital of Third Military Medical University (Army Medical University), Chongqing, 400037 China; 3 Department of Burns and Reconstructive Surgery, Jinan Military General Hospital, Jinan, 250000 China; 4 Department of Burns, The First Affiliated Hospital, SunYat-Sen University, Guangzhou, 510080 China

**Keywords:** Thermoplastic polyurethane, Porous structure, Biomimetic polydopamine, Nano-silver, Antibacterial, Methicillin-resistant *staphylococcus aureus*, Wound dressing

## Abstract

**Background:**

Developing an ideal wound dressing that meets the multiple demands of good biocompatibility, an appropriate porous structure, superior mechanical property and excellent antibacterial activity against drug-resistant bacteria is highly desirable for clinical wound care. Biocompatible thermoplastic polyurethane (TPU) membranes are promising candidates as a scaffold; however, their lack of a suitable porous structure and antibacterial activity has limited their application. Antibiotics are generally used for preventing bacterial infections, but the global emergence of drug-resistant bacteria continues to cause social concerns.

**Results:**

Consequently, we prepared a flexible dressing based on a TPU membrane with a specific porous structure and then modified it with a biomimetic polydopamine coating to prepare in situ a nano-silver (NS)-based composite via a facile and eco-friendly approach. SEM images showed that the TPU/NS membranes were characterized by an ideal porous structure (pore size: ~ 85 μm, porosity: ~ 65%) that was decorated with nano-silver particles. ATR-FITR and XRD spectroscopy further confirmed the stepwise deposition of polydopamine and nano-silver. Water contact angle measurement indicated improved surface hydrophilicity after coating with polydopamine. Tensile testing demonstrated that the TPU/NS membranes had an acceptable mechanical strength and excellent flexibility. Subsequently, bacterial suspension assay, plate counting methods and Live/Dead staining assays demonstrated that the optimized TPU/NS2.5 membranes possessed excellent antibacterial activity against *P. aeruginosa*, *E. coli*, *S. aureus* and MRSA bacteria, while CCK8 testing, SEM observations and cell apoptosis assays demonstrated that they had no measurable cytotoxicity toward mammalian cells. Moreover, a steady and safe silver-releasing profile recorded by ICP-MS confirmed these results. Finally, by using a bacteria-infected (MRSA or *P. aeruginosa*) murine wound model, we found that TPU/NS2.5 membranes could prevent in vivo bacterial infections and promote wound healing via accelerating the re-epithelialization process, and these membranes had no obvious toxicity toward normal tissues.

**Conclusion:**

Based on these results, the TPU/NS2.5 nanocomposite has great potential for the management of wounds, particularly for wounds caused by drug-resistant bacteria.

## Background

Wound dressings play a critical role in the management of cutaneous wounds because they can protect the wounds and promote the regeneration of dermal and epidermal tissues [1, 2]. Due to an increasing number of people suffering from burns, diabetic ulcers, venous ulcers etc., the demand for better dressings is growing dramatically [3, 4]. Generally, an ideal dressing should possess non-toxicity, biocompatibility, robust mechanical properties and suitable permeability for gas and water exchange [5, 6]. As natural biomaterials, collagen, gelatin, alginate and chitosan have been extensively used to prepare various types of dressings (e.g., hydrogels, films and foams) due to their biocompatibility and biodegradability [1, 7]. However, their poor mechanical properties make it difficult for them to meet rigorous clinical requirements [5, 8].

Thermoplastic polyurethane (TPU) is a biocompatible and biodegradable elastomer that has been approved by the FDA, and has been widely applied in biomedical science [9, 10]. It has been reported that TPU can be used for catheters, vascular grafts and drug delivery carriers [11–13]. Moreover, TPU also exhibits remarkable chemical stability and good mechanical properties [10, 12]. These superior performances indicate that TPU is a promising candidate for wound dressings. However, lacking antibacterial activity would limit its application in wound care, as bacterial infections always pose a severe threat to the wound bed [14].

A feasible way to solve this problem is to incorporate antibiotics such as amoxicillin, vancomycin or gentamicin into wound dressings [15–17]. Nevertheless, the emergence of drug-resistance worldwide due to the overuse of antibiotics continues to threaten public health [18]. Thus, alternative antibacterial agents are urgently needed. Nano-silver (NS) as an excellent antibacterial agent with robust and broad-spectrum bactericidal activity against both Gram-positive and Gram-negative bacteria, including multi-drug-resistant bacteria such as methicillin-resistant *staphylococcus aureus* (MRSA) [19, 20]. More importantly, it has been proposed that nano-silver destroys bacteria through various mechanisms (cell membrane disruption, DNA replication interference, respiratory function inhibition) without causing drug-resistance [21, 22]. However, the toxicity of nano-silver towards mammalian cells is a concern. Recent studies have demonstrated that the toxic effects of nano-silver occur only at high concentrations, and the incorporation of nano-silver into materials mitigates the toxicity [23, 24]. Consequently, nano-silver is considered as an ideal antibacterial agent for inclusion in biomaterials.

Although several methods have been employed to synthesize nano-silver, the complicated methods of preparation and the formation of hazardous by-products that occur during the synthetic process renders them undesirable [25–27]. Recently, biomimetic polydopamine (PD) has attracted much interest in tissue engineering due to its versatile surface modification and reducing properties [28, 29]. Inspired by the phenomenon of mussel-adhesion, researchers have discovered that dopamine (DA) undergoes self-polymerization to form a polydopamine layer under alkaline conditions. A layer of polydopamine can be coated onto any type of biomaterials, and being enriched with amines and catechols enables the in situ formation and integration of metal nanoparticles on the materials’ surface via a redox reaction. Furthermore, polydopamine is nontoxic, and no harmful by-products are produced during the mild formation process [30–32].

**Scheme 1:**
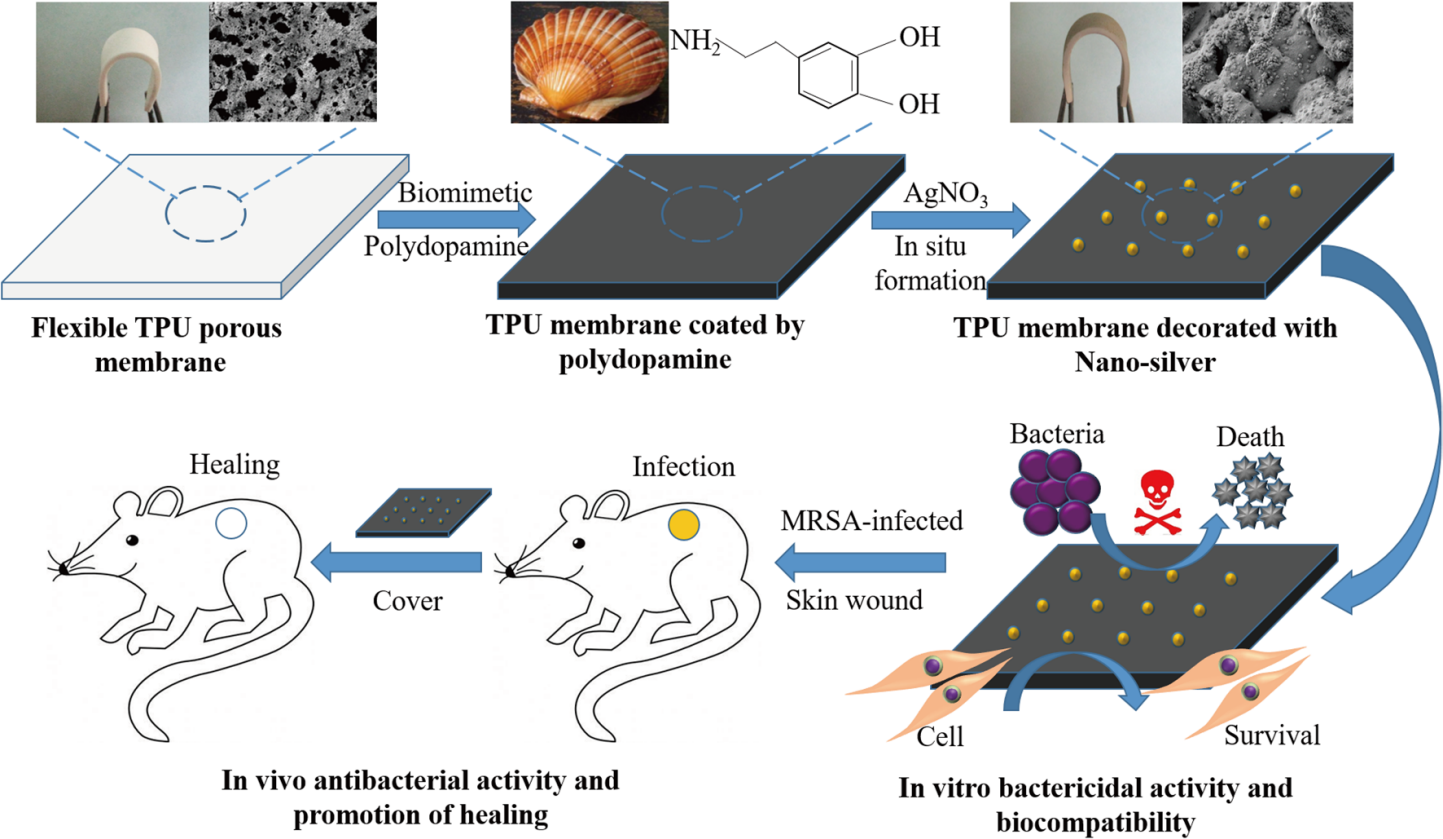
Schematic of the synthesis and the biological role of TPU/NS nanocomposites

In this study, we prepared a flexible antibacterial wound dressing of TPU/NS by a facile and green approach. A TPU porous membrane was first prepared using the combined methods of immersion precipitation and particle leaching [33], then a polydopamine layer and nano-silver were coated in situ onto the TPU membrane stepwise in an aqueous solution at room temperature (Scheme 1). Characterization of the nanocomposite was conducted by SEM, ATR-FITR, XRD, ICP-MS, water contact angle measurement and tensile testing. In vitro antibacterial activity was assessed using a bacterial suspension assay, plate counting methods and Live/Dead staining assays, while biocompatibility was evaluated using a CCK8 test, SEM observations and cell apoptosis assays. Finally, the in vivo effects of the membrane on MRSA- and *P. aeruginosa*-infected wounds and major organs were evaluated in mice. We hypothesized that this nanocomposite could provide efficient bactericidal activity without causing eukaryotic toxicity both in vitro and in vivo, and could be a novel strategy for the management of wounds.

## Materials and methods

### Materials and animals

Thermoplastic polyurethane (TPU) was obtained from Lubrizol (USA). *N,N*-dimethylformamide (DMF) was purchased from Sigma-Aldrich (MO, USA). Silver nitrate (AgNO_3_, 99%), dopamine and sodium citrate (Na-citrate) were purchased from Sangon (Shanghai, China). BALB/c mice (male, ~ 25 g) were obtained from the Experimental Animal Department of Third Military Medical University (TMMU). All animal experimental methods were approved by the ethical committee of the TMMU, and all animal experiments were performed in accordance with the guidelines of the TMMU.

### Preparation of a TPU porous membrane

A TPU porous membrane was prepared using the combined methods of immersion precipitation and particle leaching. Briefly, 12.5 g of TPU granules, 100 mL of DMF and 25 g of Na-citrate powder (particle size: 75–150 μm) were blended thoroughly to form a homogeneous TPU/DMF/Na-citrate mixture. Next, the mixture was placed in a vacuum oven for 2 h at 50 °C to remove air bubbles, and then was uniformly cast in a polytetrafluoroethylene mould, followed by immersion into ethanol. After solidification, the TPU membrane was washed with deionized water for 2 days to extract the Na-citrate particles. Finally, the membrane was dried at 45 °C for 4 h, and a microporous TPU membrane was obtained.

### Synthesis of a TPU/NS nanocomposite

To prepare a TPU/NS nanocomposite, the TPU membrane was first immersed into a dopamine (DA) solution (2 mg/mL in 10 mM Tris–HCl, pH 8.5) for 20 h at 25 °C. During this time the colour of the membrane evolved from pale white to dark brown. Subsequently, the DA-coated membrane was placed into a AgNO_3_ solution at different concentrations (1, 2.5 and 5 mM) for 6 h at 25 °C protected from light. Finally, the samples were washed with deionized water twice to remove the residual silver ions and dried at 45 °C for 4 h. The DA-coated and AgNO_3_-treated membranes were labelled as TPU/DA, TPU/NS1, TPU/NS2.5 and TPU/NS5, respectively.

### Characterization of the TPU/NS nanocomposite

The morphologies of the pristine TPU and TPU/NS membranes were observed using scanning electron microscopy (SEM, Crossbeam 340, Zeiss, Germany), and the compositional analysis was conducted by energy-dispersive X-ray spectrometry (EDS). Based on the SEM images, the average pore size, porosity and diameters of the nano-silver particles were measured using Image J software and was read by two independent investigators. X-ray diffraction (XRD) spectra of the TPU and TPU/NS membranes were obtained with a 2*θ* range between 10° and 80° using an X-ray diffractometer (X’Pert Power, Panalytical B.V., Netherlands). The attenuated total reflectance Fourier transform-infrared (ATR-FTIR) spectra of the samples were obtained using an ATR-FTIR spectrometer (Nicolet 460, USA). The water contact angles of the samples were measured using a contact angle analyser (Theta Lite 101, Biolin Scientific, Sweden).

### Mechanical properties measurements

The mechanical properties of as-prepared membranes were measured using a tensile test as previously described [5]. Briefly, the dumbbell-shaped specimens were gripped and oriented vertically with metallic clamps. Then, the specimens were stretched to failure at a constant speed of 20 mm/min using an MTS industrial testing system (Exceed 44, Germany) at room temperature. Three specimens were used for each group.

### Antibacterial assay

The antibacterial activity of TPU/NS nanocomposites towards *Pseudomonas aeruginosa* (*P. aeruginosa*, ATCC 27853), *Escherichia coli* (*E. coli*, ATCC 25922), *Staphylococcus aureus* (*S. aureus*, ATCC 25923), and MRSA (ATCC 43300) was determined using a bacterial suspension assay [34]. Briefly, a bacterial colony was incubated in 4 mL of Luria–Bertani (LB) medium with shaking at 37 °C overnight to obtain a log-phase bacterial suspension. After diluting with LB medium to the starting concentration (optical density at 600 nm; OD_600_ = 0.07), 500 μL of the bacterial suspension were added to each well of a 24-well plate. Next, a sterilized test membrane (10 × 10 mm) was immersed into the bacterial suspension. The plate was placed in a shaker and incubated at 37 °C for 24 h at 50 rpm. Finally, 100 μL of bacterial suspension from each well was transferred into a 96-well plate, and the OD_600_ was recorded using an enzyme-linked immunosorbent assay reader. To quantify the number of bacteria, the bacterial suspension was serially diluted with saline to obtain a 2500-fold dilution for *P. aeruginosa* and *E. coli*, or a 10000-fold dilution for *S. aureus* and MRSA, and 25 μL of the diluted bacterial solution was uniformly spread on agar plate. After incubating at 37 °C for 18 h, the bacterial colonies were photographed and the number of bacterial colonies was counted. In this assay, a bacterial solution without any treatment served as a control group.

In addition, the antibacterial activity of samples against *P. aeruginosa* and MRSA was further evaluated qualitatively using a Live/Dead™ Baclight™ Bacterial Viability Kit (Invitrogen, USA). The green-fluorescing SYTO9 and red-fluorescing propidium iodide (PI) in the kit can label live and dead bacteria, respectively [35, 36]. Briefly, the bacterial suspension after 24 h of co-incubation was harvested and washed with phosphate-buffered saline (PBS) twice, then 5 μL of SYTO9 and 5 μL of PI dye were added to 1 mL of bacterial solutions and incubated at room temperature for 15 min in the dark. Finally, the stained bacteria were washed two times with PBS and then visualized using a fluorescence microscope (Olympus, Japan). To determine the long-term antibacterial activity of the TPU/NS, samples were incubated with bacterial suspensions for 14 days at 37 °C and then stained using the Live/Dead™ Baclight™ Bacterial Viability Kit as described above.

### In vitro evaluation of cytotoxicity

The cytotoxicity of as-prepared membranes against HaCaT cells and NIH3T3 fibroblasts was investigated according to ISO 10993-5. First, the leach liquor was obtained as follows: sterilized specimens (1.4-cm diameter) were immersed into 1 mL of RPMI1640 or DMEM medium at 37 °C for 24 h. The medium was then sterilized by filtration using an aseptic filter (pore size: 0.22 μm). The leach liquor in RPMI1640 medium was used to treat HaCaT cells, while that in DMEM medium was used to treat NIH3T3 fibroblasts. Next, 3000 HaCaT cells or NIH3T3 fibroblasts were inoculated into each well of a 96-well plate and incubated for 24 h at 37 °C. The medium was then replaced with 500 μL of leach liquor or pristine medium containing 10% foetal bovine serum (Gibco, USA). Cells inoculated with pristine medium were used as a control group. After 24 and 48 h, the medium was replaced with 100 μL of pristine medium containing 10 μL of CCK8 solution (Dojindo Laboratories, Kumamoto, Japan) and then incubated at 37 °C for 2 h. Finally, the optical density at 450 nm was measured using an enzyme-linked immunosorbent assay reader, and the cell viability was calculated.

Additionally, the cytotoxicity of samples at 48 h was determined further using SEM observations and cell apoptosis assays. For SEM observations, the cells that were treated for 48 h as described above were dehydrated with an ethanol solution and sputter-coated with gold. For cell apoptosis assays, all adherent and suspended cells were carefully harvested, and the test was performed using an Annexin V-Propidium iodide (PI) apoptosis detection kit according to the manufacturer’s protocol. Finally, the percentage of apoptotic and necrotic cells were detected using a fluorescence-activated cell sorting (FACS) sorter by an Attune Acoustic Focusing Cytometer (Life Technologies, USA), and the data were analysed using FlowJo software (Tree Star Incorporation, USA).

### Release of silver ions (Ag^+^) into PBS

To investigate the amount of Ag^+^ released from the TPU/NS2.5 membrane, a section of sterilized sample (10 × 10 mm) was immersed into 6 mL of phosphate-buffered saline (PBS) at 37 °C for different times (1, 3, 5, 7, 10 and 20 days). Subsequently, the total supernatant was collected at each determined time point and analysed by inductively coupled plasma mass spectrometry (ICP-MS, 7700× Agilent, USA). In addition, the total silver content of the membrane was determined by sampling (*n* = 3) in 4% nitric acid.

### In vivo animal study

An infectious murine full-thickness skin wound model was used for the in vivo study as previously described [37]. Briefly, mice were anesthetized by intraperitoneal injection of 1% pentobarbital, and their dorsal surface was shaved. Then, two 6 mm diameter wounds were created on the dorsal skin and photographed to represent the initial wound area. Afterwards, 10 μL of MRSA or *P. aeruginosa* bacterial suspensions that were standardized to 0.5 McFarland standards (10^8^ CFU/mL) were placed at the centre of the wound. Sterilized samples (10 × 10 mm) of Vaseline gauze, TPU, TPU/DA and TPU/NS 2.5 were then placed over the bacteria-seeded wounds and fixed in place using an adhesive biological membrane. The wound that was not treated with bacteria served as Blank group. Five mice were used in each group. On post-surgery days 1, 3 and 7, the cover membranes were removed and the wounds were photographed. The wound areas at different times were meticulously measured using IPP 6.0 software by two independent researchers and the percentage of closed wound area was calculated using the following formula:

$$ \% {\text{ of closed wound area}}\, = \,\left( {I\, - \,R} \right)/I\, \times \, 100\% . $$

where *I* indicates the number of pixels in the initial wound area, and *R* indicates the number of pixels in the unhealed wound area at the determined time.

Furthermore, the wound tissues (6 × 6 mm) on post-surgery day 7 in each group were harvested and homogenized in 5 mL of physiological saline, and the number of bacteria was counted using the colony counting method as described above.

In addition, five additional mice were observed in each group until the wounds were completely healed to determine the average number of days for complete wound closure.

### Histological examinations

At day 7 post-surgery, the wound samples including the entire wound with adjacent normal skin (10 × 10 mm) were collected and fixed in 4% paraformaldehyde. After 24 h, the samples were sectioned and stained with hematoxylin and eosin (H&E) for histological analysis. Based on these H&E stained sections, the number of inflammatory cells infiltrated in the wound edge, and the length of the newly regenerated epidermis, which was defined as the distance from the border between normal skin and the wound region to the anterior edges of the newly regenerated epidermis [37], were measured using Image J software by two independent pathologists.

In addition, to investigate the in vivo toxicity of TPU/NS2.5, another five mice (without bacterial treatment) in each group were sacrificed at day 7, and the major organs, including heart, liver, spleen, lung and kidney, were harvested for H&E staining.

### Statistical analysis

The experimental data were expressed as the means ± standard deviations (SD) and statistically compared using one-way ANOVA. The statistical significance was set as *p* < 0.05 (“*”) and *p* < 0.01 (“**”), and *ns* indicates no significant differences.

## Results and discussion

### Morphology and characterization of TPU/NS nanocomposites

**Fig. 1:**
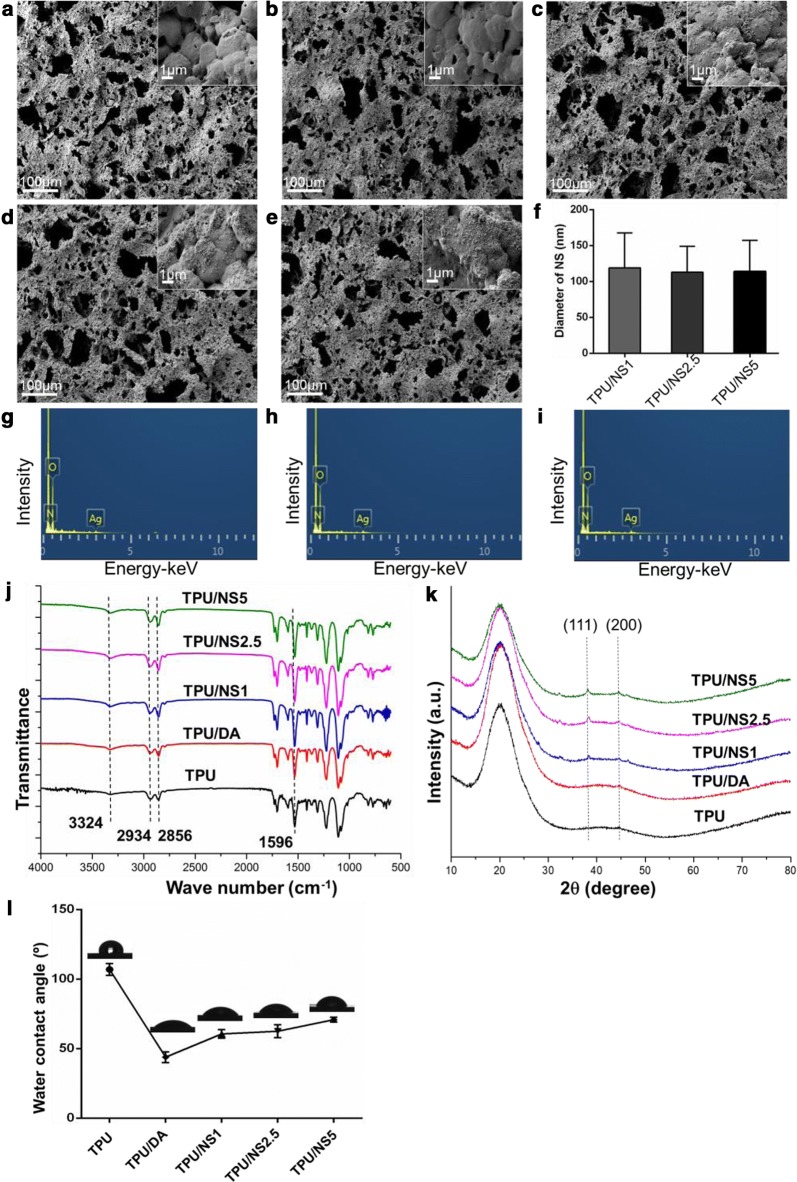
Morphology of **a** TPU, **b** TPU/DA, **c** TPU/NS1, **d** TPU/NS2.5 and **e** TPU/NS5 membranes. The insets indicate the magnification of the membranes’ surface. **f** Average diameter of nano-silver that formed on the surface of TPU/NS membranes. EDS spectra of **g** TPU/NS1, **h** TPU/NS2.5 and **i** TPU/NS5 membranes. **j** ATR-FITR spectra, **k** XRD spectra, and **l** water contact angles of as-prepared membranes

The structure and morphologies of pristine TPU and TPU/NS membranes were observed using SEM. As shown in Fig. 1a–e, all of the membranes were characterized by a similar microporous structure, indicating that polydopamine coating and nano-silver anchoring had no obvious influence on the integrity of pristine TPU membranes. Nevertheless, as shown in the magnified insets (Fig. 1c–e), many nanoparticles were uniformly decorated on the surface of the TPU membrane after immersion in a AgNO_3_ solution, and EDS analysis demonstrated the existence of elemental Ag (Fig. 1g–i), indicating the successful in situ formation of nano-silver on the membrane as a result of a reductive effect of polydopamine. These observations indicate that a nanocomposite of a TPU porous membrane integrated with nano-silver was successful prepared as expected. Moreover, to further illustrate the features of such porous structures, the average pore size, porosity and pore density of TPU/NS membranes were measured using Image J software, and the results are listed in Table 1. According to a previous study, TPU/NS membranes with large pores (> 80 μm) would be beneficial for cell adhesion and proliferation [38]. Furthermore, the TPU/NS membranes had high porosity (> 64%), which is favourable for gas and water exchange and promoting wound healing [33].

**Table 1:** The average pore size, porosity and pore density of TPU, TPU/DA, TPU/NS1, TPU/NS2.5 and TPU/NS5 membranes

	Pore size (μm)	Porosity (%)	Pore density (pores/mm^2^)
TPU	84.1 ± 12.3	69.5 ± 3.6	88.9 ± 7.2
TPU/DA	82.3 ± 11.9	67.0 ± 6.6	89.8 ± 12.2
TPU/NS1	85.5 ± 18.4	65.4 ± 5.2	83.3 ± 6.9
TPU/NS2.5	85.0 ± 16.9	65.4 ± 5.6	84.3 ± 9.5
TPU/NS5	83.3 ± 10.6	64.8 ± 5.0	85.2 ± 6.4

The chemical structures of the prepared membranes were determined using ATR-FTIR analysis. As shown in Fig. 1j, characteristic absorption peaks of TPU were observed at 3324 cm^−1^, attributed to the N–H group, and 2934 and 2856 cm^−1^, attributed to the –CH_2_ group [39]. The spectrum of TPU/DA was similar to that of TPU, but the peak at approximately 3324 cm^−1^ became stronger after coating with polydopamine, which was attributed to the stretching vibration band of the –OH group [23]. Compared with TPU/DA, the TPU/NS spectra showed intense absorption peaks at 1596 and 3324 cm^−1^, which might be attributed to the existence of nano-silver in the composite [40]. The XRD results further verified the in situ formation of nano-silver. As shown in Fig. 1k, compared with TPU and TPU/DA membranes, two characteristic peaks at 2*θ* = 38.1° and 44.4° were obviously present in the diffractogram of TPU/NS membranes, and these corresponded to the (111) and (200) planes of silver nanoparticles, respectively (JCPDS No. 65-2871) [41].

Surface wettability is an important parameter that can influence the biological behaviour of biomaterials, therefore, the water contact angles of TPU, TPU/DA, TPU/NS1, TPU/NS2.5 and TPU/NS5 were measured. Figure 1l showed the water contact angle of pristine TPU to be 106.9°, indicating the intrinsic hydrophobic nature of TPU. After coating with polydopamine, the water contact angles of TPU/DA, TPU/NS1, TPU/NS2.5 and TPU/NS5 were dramatically decreased to 43.9°, 60.6°, 62.5° and 70.8°, respectively, which was consistent with previous studies that indicated that polydopamine coatings yielded hydrophilic surfaces [42]. The improvement of hydrophilicity on the membrane surface is helpful for promoting cell adhesion [28]. It should be noted that the water contact angles of TPU/NS membranes were higher than that of TPU/DA membranes, and this was probably attributed to the increased surface roughness after deposition of nano-silver [43]. Taken together, the SEM, EDS, ATR-FTIR, XRD and water contact angle results confirmed the successful preparation of TPU/NS nanocomposites.

### Mechanical properties of TPU/NS nanocomposites

**Fig. 2:**
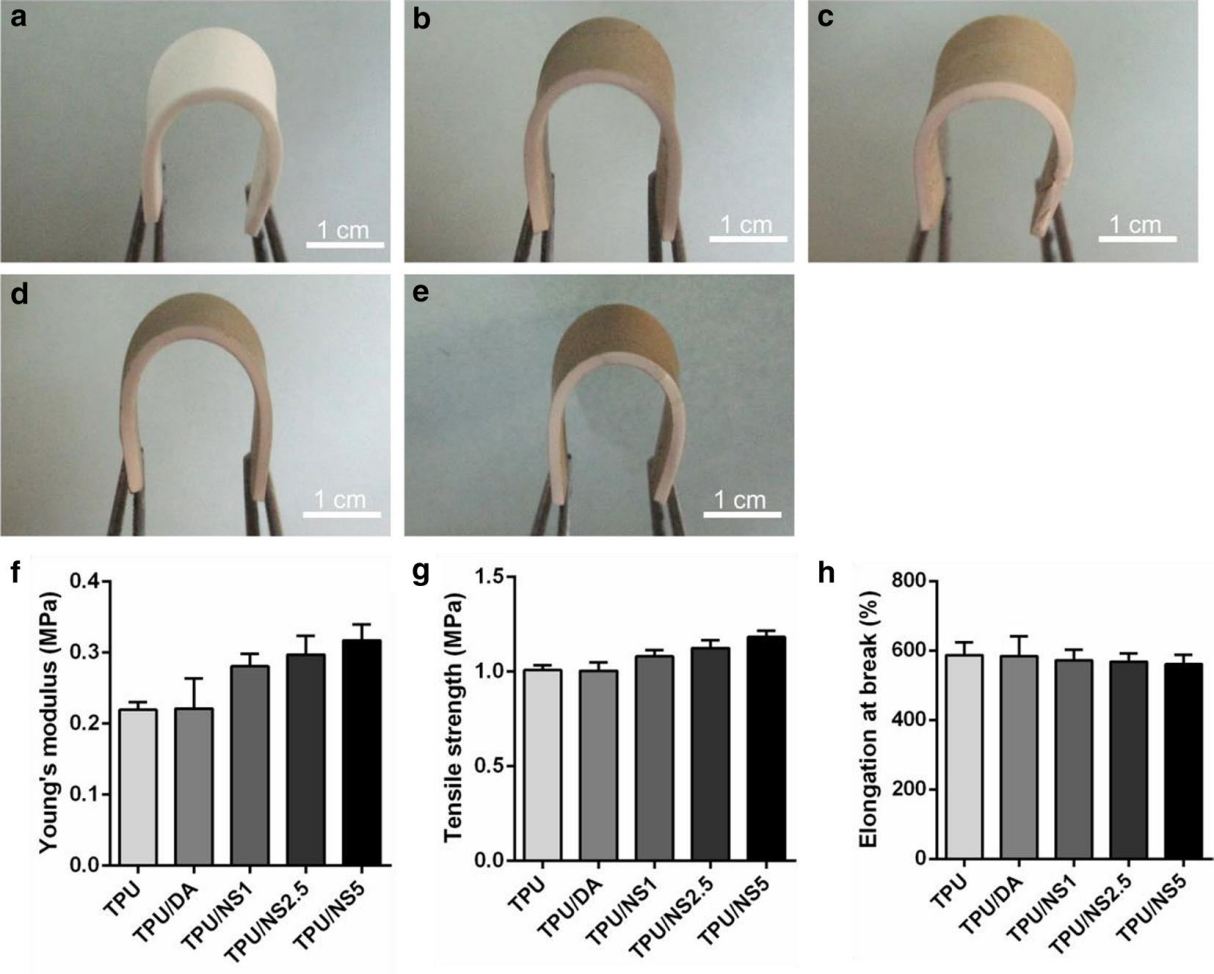
Images showing bending of the **a** TPU, **b** TPU/DA, **c** TPU/NS1, **d** TPU/NS2.5 and **e** TPU/NS5 membranes. Mechanical properties of as-prepared membranes: **f** Young’s modulus, **g** Tensile strength and **h** elongation at break (%)

An ideal wound dressing should possess excellent flexibility and mechanical strength so that it can protect wounds from physical damage and resist deformation caused by rubbing or collision [44, 45]. The photographs in Fig. 2a–e demonstrate that the elasticity and flexibility of TPU were well-preserved in TPU/NS membranes after nano-silver deposition, which suggested that the nanocomposites could adequately cover the wounds and effectively avoid secondary damage [46]. The mechanical properties of TPU and TPU/NS membranes were further evaluated using a tensile test. As shown in Fig. 2f, the Young’s modulus of pristine TPU and TPU/DA were 0.22 ± 0.01 MPa and 0.22 ± 0.04 MPa, respectively, while that of TPU/NS1, TPU/NS2.5 and TPU/NS5 were increased to 0.28 ± 0.02 MPa, 0.30 ± 0.03 MPa and 0.32 ± 0.02 MPa, respectively. Similarly, the tensile strengths of nano-silver-decorated TPU/NS composite membranes were higher than TPU and TPU/DA membranes and performed in a silver dose-dependent manner (Fig. 2g). These results indicated that compared with pristine TPU, the TPU/NS composite membranes had stronger mechanical strength and stiffness, which might be attributed to the reinforcement effect of nano-silver as has been previously reported [47]. In addition, the percentage of elongation at break for all membranes was in a range of 561–587% (Fig. 2h), demonstrating that TPU/NS composite membranes still possessed superior ductility and flexibility as shown in the photographs (Fig. 2a–e). More noteworthy, compared with the mechanical properties of commercial Kaltostat™ dressings (tensile strength: 0.9 MPa; elongation at break: 10.8%; Young’s modulus: 1.3 MPa), TPU/NS composite membranes exhibited a higher tensile strength and percent elongation at break and lower Young’s modulus, indicating that our nanocomposites were flexible, comfortable and resilient [48]. Collectively, the synthesized TPU/NS composite membranes with excellent mechanical performances could be valuable candidates for wound dressings.

### Antibacterial activity of TPU/NS nanocomposites

**Fig. 3:**
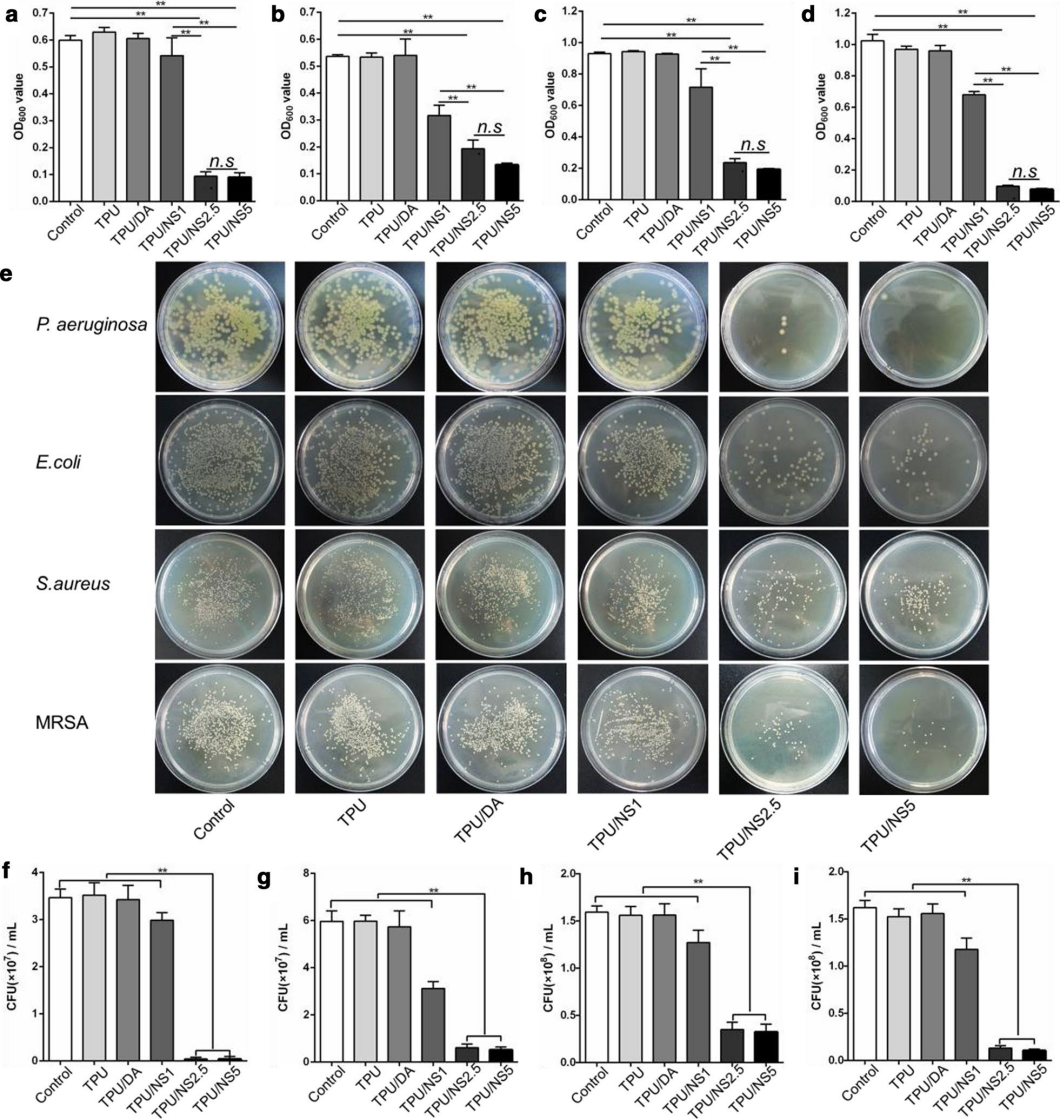
In vitro antibacterial activity of the TPU, TPU/DA, TPU/NS1, TPU/NS2.5 and TPU/NS5 membranes. OD_600_ values of **a** *P. aeruginosa*, **b** *E. coli*, **c** *S. aureus* and **d** MRSA bacterial suspensions after 24 h co-incubation with samples. **e** Photographs of bacterial colonies of *P. aeruginosa*, *E. coli*, *S. aureus* and MRSA. The number of bacterial colonies formed by **f** *P. aeruginosa*, **g** *E. coli*, **h** *S. aureus* and **i** MRSA. The values are shown as the mean ± SD (*n* = 3)

**Fig. 4:**
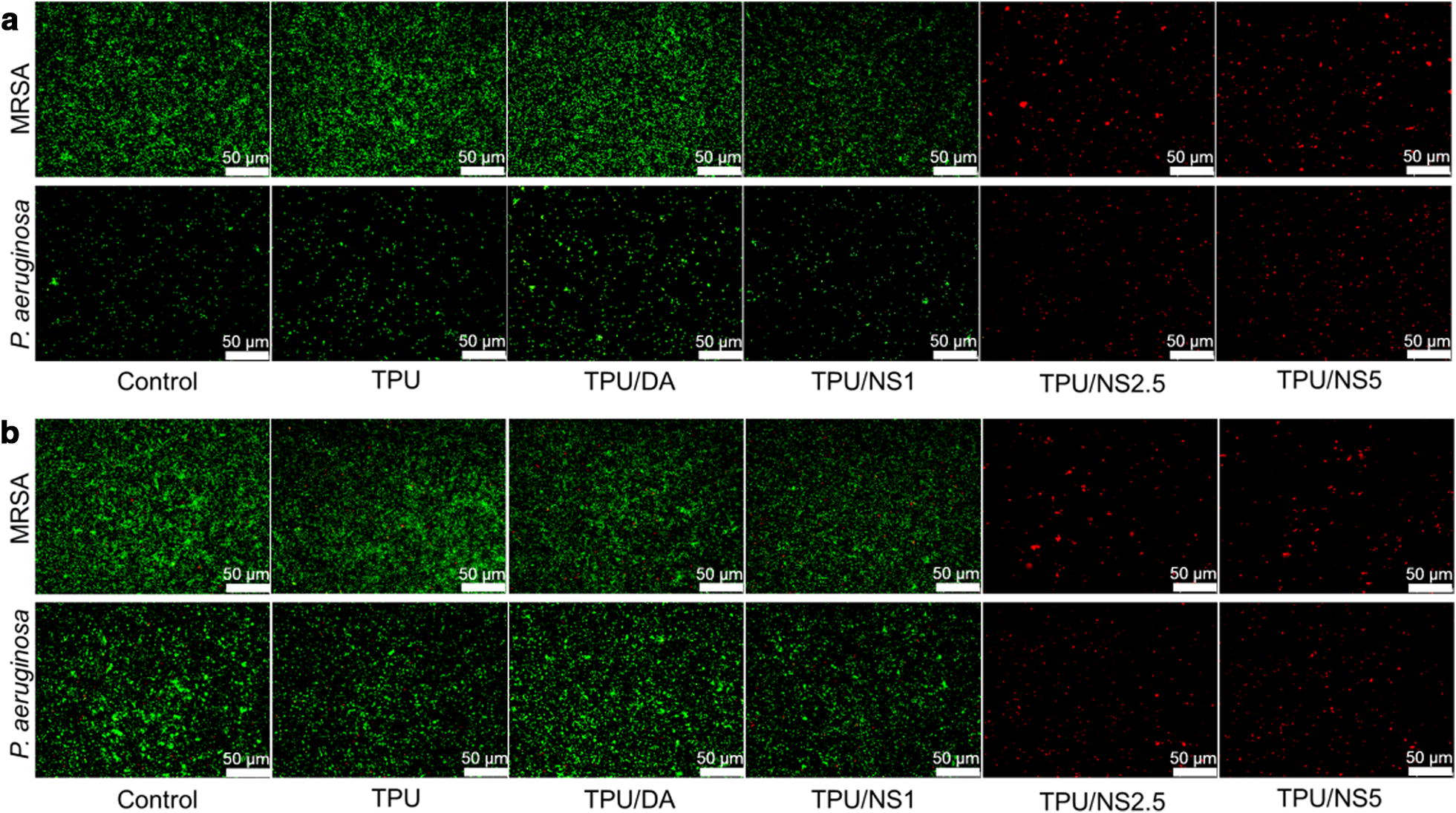
Fluorescence microscopic images of MRSA and *P. aeruginosa* in different groups after **a** 24 h and **b** 14 days of incubation. MRSA and *P. aeruginosa* cells were stained using a Live/Dead™ Baclight™ Bacterial Viability Kit

The in vitro antibacterial activity of TPU/NS nanocomposite against Gram-negative *P. aeruginosa* and *E. coli* and Gram-positive *S. aureus* and MRSA bacteria was first determined using a bacterial suspension assay. Figure 3a–d showed the OD_600_ values of bacterial suspensions that were co-incubated with or without test samples after 24 h. It was observed that the OD_600_ values in the TPU and TPU/DA groups were similar to those in the control group, while the OD_600_ values for all four bacterial strains were decreased in a dose-dependent manner after treatment with TPU/NS membranes. Specifically, the OD_600_ values were significantly lower in the TPU/NS2.5 and TPU/NS5 groups than that in the other groups. These results indicated that TPU/NS2.5 and TPU/NS5 membranes had acceptable broad-spectrum antibacterial activity due to the effect of the deposited nano-silver. In addition, and consistent with the OD values, the number of bacterial colonies formed by the co-incubated bacterial suspension were also significantly less in the TPU/NS2.5 and TPU/NS5 groups than that in the other groups (Fig. 3e–i), demonstrating that these two nanocomposites could efficiently inhibit bacterial growth. To further evaluate the antibacterial activity of TPU/NS membranes against Gram-positive MRSA and Gram-negative *P. aeruginosa*, a Live/Dead staining assay was conducted. In this assay, live bacterial cells with intact membranes were stained green, while dead bacterial cells with damaged membranes were stained red [35, 36]. As shown in Fig. 4a, many live cells were observed in the control, TPU, TPU/DA and TPU/NS1 groups both with MRSA and *P. aeruginosa* after 24 h of incubation, whereas an abundance of bacteria in TPU/NS2.5 and TPU/NS5 groups were dead. This phenomenon was similar to the results of the bacterial suspension assay (Fig. 3), which further confirmed that TPU/NS2.5 and TPU/NS5 had satisfactory bactericidal activity. Additionally, to investigate the long-term antibacterial activity of TPU/NS2.5 and TPU/NS5, bacterial suspensions were continuously incubated with samples for 14 days and then detected using the Live/Dead staining assay. The fluorescence images in Fig. 4b revealed that many of the MRSA and *P. aeruginosa* bacteria were still alive in the control, TPU, TPU/DA and TPU/NS1 groups, while few live cells were observed in the TPU/NS2.5 and TPU/NS5 groups. This result indicated that TPU/NS2.5 and TPU/NS5 membranes could persistently inhibit the growth of bacteria, which further demonstrated their excellent antibacterial activity. Although the antibacterial mechanism for nano-silver is not fully understood, we presumed that our TPU/NS nanocomposite could release Ag^+^ to combat bacteria in solution and destroy them via damaging their cell membranes, interfering with DNA replication and inhibiting the activity of respiratory enzymes, as reported in previous studies [21, 26]. Overall, TPU/NS2.5 and TPU/NS5 membranes displayed excellent antibacterial performance against microorganisms including multi-drug-resistant bacteria, suggesting their potential application for treating infected wounds in vivo.

### Cytotoxicity of TPU/NS nanocomposites

**Fig. 5:**
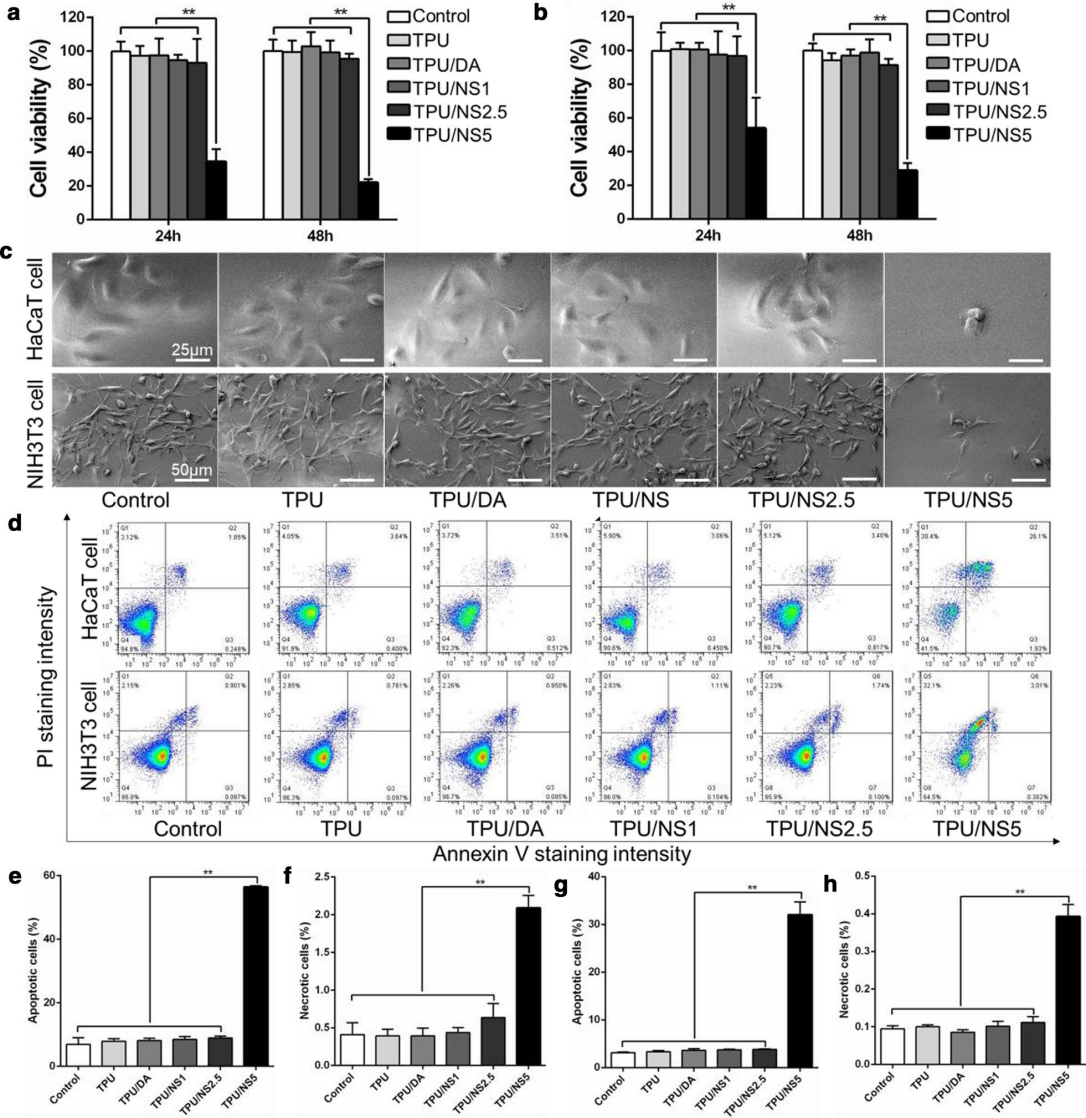
Cytotoxicity evaluation of membranes. Cell viability of **a** HaCaT cells and **b** NIH3T3 fibroblasts treated with leach liquor extracted from samples after 24 and 48 h. Corresponding cell morphology of **c** HaCaT cells and NIH3T3 fibroblasts after 48 h. **d** FACS results revealing the distribution of apoptotic and necrotic cells. Quantitative data for the percentage of **e** apoptotic cells and **f** necrotic cells for HaCaT cells, and the percentage of **g** apoptotic cells and **h** necrotic cells for NIH3T3 fibroblasts in each group. The values are shown as the mean ± SD (*n* = 3)

Biosafety is a crucial factor for a wound dressing, therefore we meticulously investigated the cytotoxicity of TPU/NS membranes using a CCK8 test, SEM observations and cell apoptosis assays. Because keratinocytes and fibroblasts are the main components of cutaneous tissues, HaCaT cells and NIH3T3 fibroblasts were chosen as the cell models for this assay. As shown in Fig. 5a, b, both the HaCaT cells and NIH3T3 fibroblasts that were treated with leach liquor that was extracted from TPU, TPU/DA, TPU/NS1 and TPU/NS2.5 showed similar cell viabilities (> 90%) as that in the control group after 24 h and 48 h, revealing that these membranes exhibited no obvious cytotoxicity. However, the cell viabilities of both HaCaT cells (33.4% and 22.0% after 24 h and 48 h, respectively) and NIH3T3 fibroblasts (54.0% and 28.9% after 24 h and 48 h, respectively) in the TPU/NS5 group were significantly lower than those in the other groups, indicating that TPU/NS5 exhibited strong toxicity towards mammalian cells. Moreover, the SEM images also showed that TPU/NS1 and TPU/NS2.5-treated HaCaT cells and NIH3T3 fibroblasts spread as well as untreated cells, while the growth of cells in the TPU/NS5 group were obviously restrained (Fig. 5c), which was probably attributed to the high dose of nano-silver that could not only kill bacteria but also impair eukaryotes [24].

To further evaluate the cytotoxicity of TPU/NS membranes, a flow cytometry apoptosis assay was employed and the results were shown in Fig. 5d–h. The Annexin V^+^ quadrants (both for the PI^+^ and PI^−^) represent apoptotic cells, while the PI^+^ and Annexin V^−^ quadrant represent necrotic cells [49]. For HaCaT cells (Fig. 5e, f), the percentages of apoptotic and necrotic cells in the control, TPU, TPU/DA, TPU/NS1 and TPU/NS2.5 groups were all below 9.0% and 0.7%, respectively, and no significant differences were observed among these groups, confirming that TPU/NS1 and TPU/NS2.5 were biocompatible with mammalian cells. Nevertheless, the corresponding ratios in the TPU/NS5 group were increased significantly to 56.4% and 2.1%, respectively. Similarly, the percentages of apoptotic (32.0%) and necrotic cells (0.4%) in TPU/NS5-treated NIH3T3 fibroblasts were also obviously higher than in the other groups (apoptotic cells: < 4.0%, necrotic cells: < 0.2%, Fig. 5g, h). These data are in agreement with the CCK8 results and the SEM observations, indicating that TPU/NS5 exhibited cytotoxicity towards HaCaT cells and NIH3T3 fibroblasts, which could be attributed to the high dose of nano-silver that induced excessive production of reactive oxygen species and caused DNA damage, leading to cell apoptosis and necrosis [50].

Based on the antibacterial activity results and the cytotoxicity evaluations, the TPU/NS2.5 membrane was selected as the optimal sample for use in subsequent experiments due to its excellent antibacterial activity and good biocompatibility.

### Ag^+^ release into PBS

**Fig. 6:**
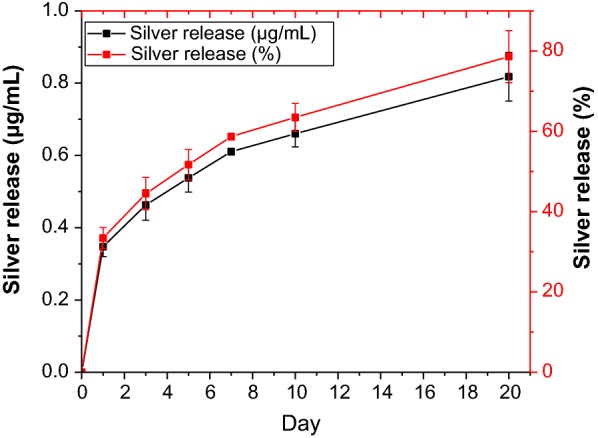
Ag^+^ released from TPU/NS2.5 membranes at days 1, 3, 5, 7, 10 and 20. The values are shown as the mean ± SD (*n* = 3)

The biological role of nano-silver was believed to be mainly dependent on the amount of Ag^+^ that was released, therefore the Ag^+^-releasing profile for TPU/NS2.5 membranes was investigated using IPC-MS [51]. The total silver content was 6.25 ± 0.93 μg/cm^2^. As shown in Fig. 6, a burst release of Ag^+^ was observed at day 1, and the measured dose of Ag^+^ (0.35 μg/mL) was much higher than the effective bactericidal dose (0.05 μg/mL) [52]. This observation agrees with the results of the in vitro antibacterial activity evaluations (Figs. 3 and 4) that TPU/NS2.5 had remarkable bactericidal capacity, further suggesting that TPU/NS2.5 could efficiently eliminate bacteria effectively when applied as a cover to infected wounds. Furthermore, a sustained release of Ag^+^ could last up to 20 days (approximately 80% of the total content), implying that TPU/NS2.5 with favourable durability could persistently protect the wounds from bacterial infections. More noteworthy, the total amount of Ag^+^ released by day 20 (< 1 μg/mL) was far less than the dose that is toxic to humans (10 μg/mL) [53], which further indicated that TPU/NS2.5 had good biocompatibility as described above (Fig. 5). These results suggest that TPU/NS2.5 membranes could effectively prevent bacterial infections in vivo and not exhibit human toxicity.

### In vivo effect of TPU/NS2.5 on healing of infected wounds

**Fig. 7:**
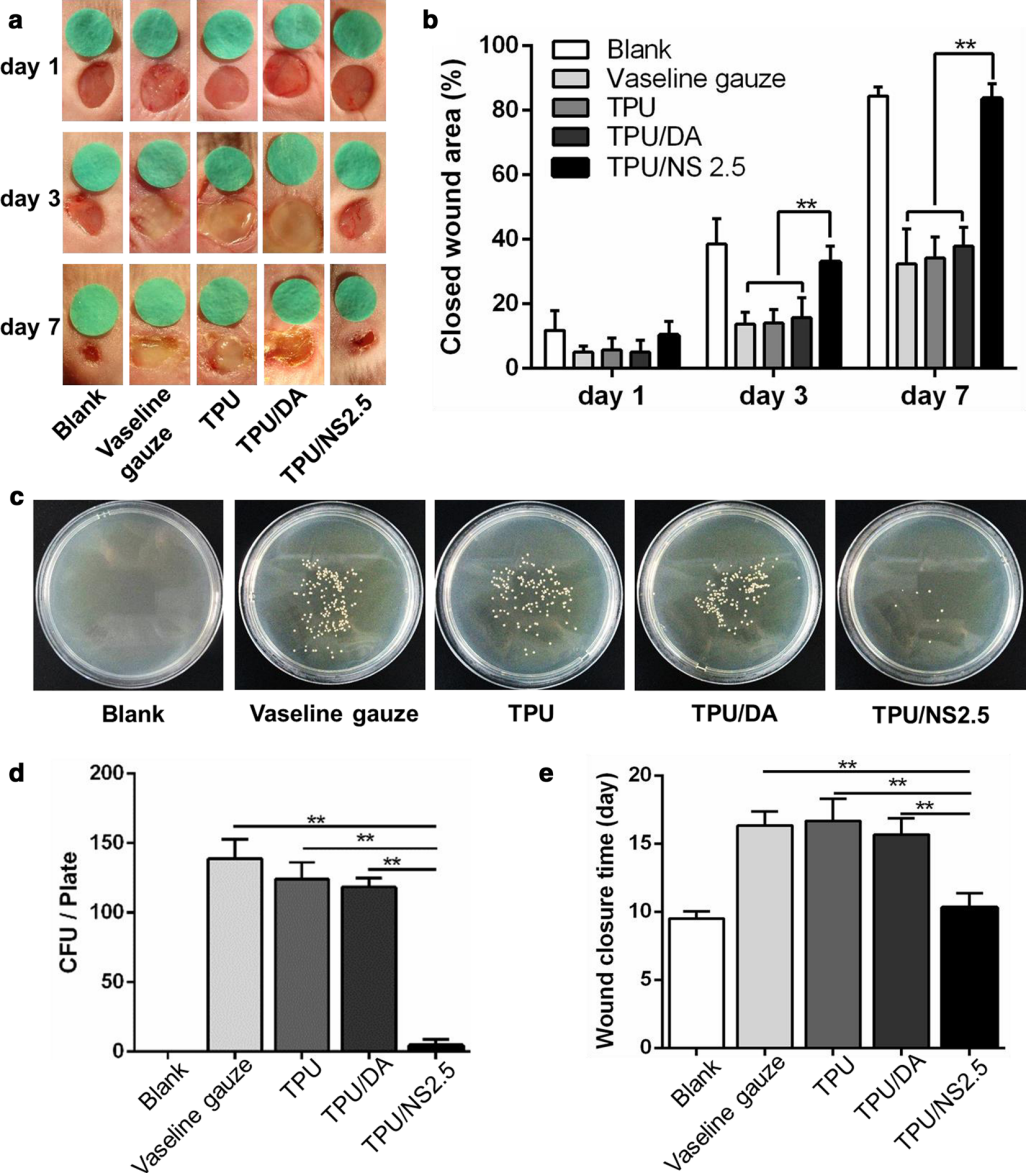
The effect of TPU/NS2.5 on the healing of MRSA-infected wounds. **a** The macroscopic appearance of wounds from the Blank, Vaseline gauze, TPU, TPU/DA and TPU/NS2.5 groups. **b** The percentage of closed wound areas at days 1, 3 and 7 post-surgery. **c** Photographs and **d** quantitative counts of bacterial colonies formed by MRSA obtained from wound tissues. **e** Average days for wound closure. The values are shown as the mean ± SD (*n* = 5)

**Fig. 8:**
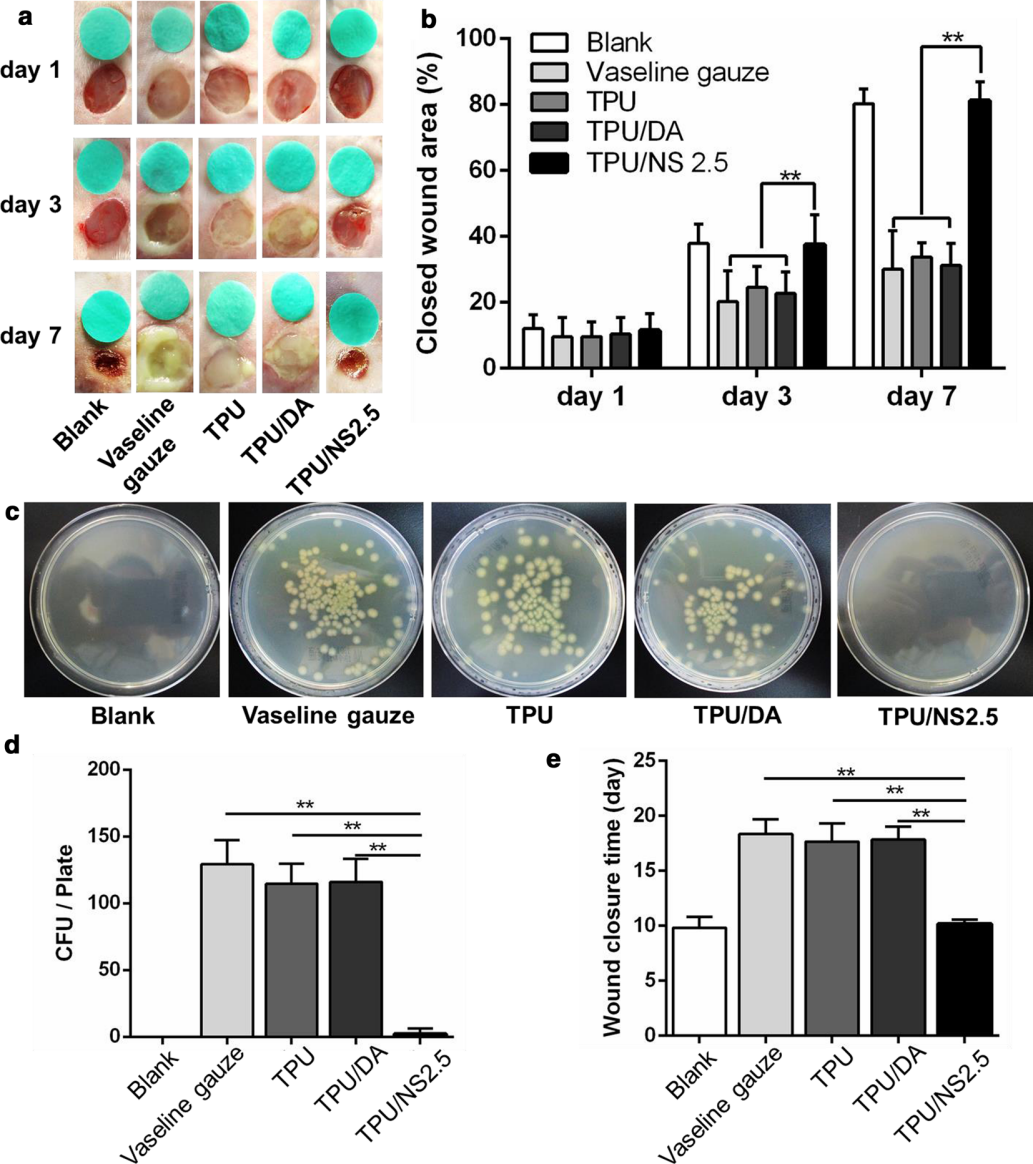
The effect of TPU/NS2.5 on the healing of *P. aeruginosa*-infected wounds. **a** The macroscopic appearance of wounds from the Blank, Vaseline gauze, TPU, TPU/DA and TPU/NS2.5 groups. **b** The percentage of closed wound areas at days 1, 3 and 7 post-surgery. **c** Photographs and **d** quantitative counts of bacterial colonies formed by *P. aeruginosa* obtained from wound tissues. **e** Average days for wound closure. The values are shown as the mean ± SD (*n* = 5)

To further investigate the effect of TPU/NS2.5 on infected wounds in vivo, a bacteria-infected (MRSA or *P. aeruginosa*) murine skin wound model was utilized. Figures 7a, b and 8a, b showed the appearance of wounds and quantitative closed wound areas in Blank (without MRSA or *P. aeruginosa*), Vaseline gauze, TPU, TPU/DA and TPU/NS2.5 groups. At day 1 post-surgery, no obvious difference was observed in the closed wound area among all the groups for both MRSA- or *P. aeruginosa*-infected wounds. However, it was apparent that abundant yellow purulent fluids permeated the wounds in the Vaseline gauze, TPU and TPU/DA groups at day 3 post-surgery, and the ambient skin tissue were obviously red and swollen, which indicated that severe infections developed. In contrast, the appearance of wounds covered with the TPU/NS2.5 membrane was as clean and dry as that in the Blank group. After 7 days of treatment, the wound in the TPU/NS2.5 group appeared to be completely healed, while festered wounds persisted in the Vaseline gauze, TPU and TPU/DA groups, demonstrating the excellent therapeutic efficacy of TPU/NS2.5 membranes in treating infected wounds. The quantitative data for the closed wound area also supported these results. At days 3 and 7 post-surgery, the percentage of closed wound areas in the TPU/NS2.5 group was significantly larger than that in the Vaseline gauze, TPU and TPU/DA groups, however, no significance difference was detected between the TPU/NS2.5 and Blank groups. Specifically, for the MRSA-infected wounds, the percentage of closed wound areas in the Blank and PVDF/NS25 groups was greater than 83% at day 7 post-surgery, while the Vaseline gauze, TPU and TPU/DA groups the percentages were 32.3%, 34.2% and 37.9%, respectively (Fig. 7b). For the *P. aeruginosa*-infected wounds, the percentage of closed wound areas in the Blank and PVDF/NS25 groups was greater than 80% at day 7 post-surgery, while in the Vaseline gauze, TPU and TPU/DA groups they were 30.1%, 33.8% and 31.2%, respectively (Fig. 8b). Simultaneously, to evaluate the actual bactericidal effect of TPU/NS2.5 in vivo, the wound tissues were harvested and homogenized to quantify the number of bacteria. As shown in Figs. 7c, d and 8c, d, compared with the Vaseline gauze, TPU and TPU/DA groups, significantly fewer bacterial colonies were found in the TPU/NS2.5 group, both for the MRSA- or *P. aeruginosa*-infected wounds, which confirmed the outstanding antibacterial activity of TPU/NS2.5 membranes in vivo. Additionally, we found that application of TPU/NS2.5 membranes significantly shortened the wound closure time. For the MRSA-infected wounds, Fig. 7e showed that the average wound closure timfor the Vaseline gauze, TPU and TPU/DA groups were 16.3, 16.7 and 15.7 days, respectively; however, it only required 10.3 days for TPU/NS2.5-treated mice, which was close to the time required for healing in the Blank group (9.5 days). For the *P. aeruginosa*-infected wounds, the average wound closure times for the Vaseline gauze, TPU and TPU/DA groups were 18.3, 17.7 and 17.8 days, respectively, while it only required 10.2 and 9.8 days for the TPU/NS2.5 and Blank groups, respectively (Fig. 8e). Taken together, these data indicated that TPU/NS2.5 could efficiently eliminate MRSA and *P. aeruginosa* infections in vivo, leading to rapid wound healing.

**Fig. 9:**
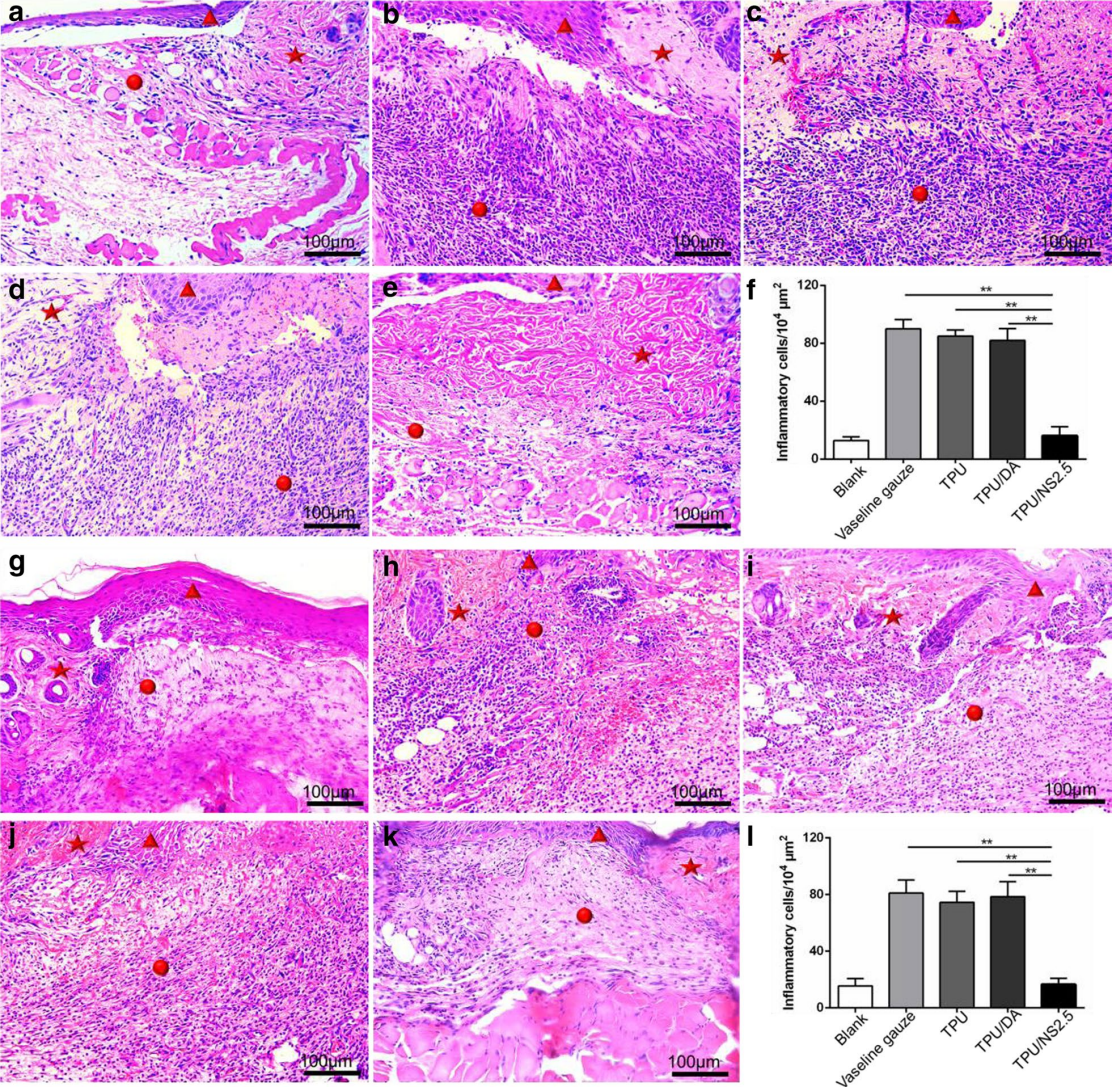
The effect of TPU/NS2.5 on inflammation. Representative H&E staining images of the **a**, **g** Blank, **b**, **h** Vaseline gauze, **c**, **i** TPU, **d**, **j** TPU/DA and **e**, **k** TPU/NS2.5 groups. The images **a**–**e** indicate MRSA-infected wounds, and the images **g**–**k** indicate *P. aeruginosa*-infected wounds. The red pentagram indicates unwounded skin tissue, the red circle indicates the wound area, and the red triangle indicates the regenerated epidermis. Quantitative counts of inflammatory cells infiltrated in the wound region of (**f**) MRSA-infected wounds and (**l**) *P. aeruginosa*-infected wounds. The values are shown as the mean ± SD (*n* = 5)

To further evaluate the wound healing process, the wound tissue at day 7 post-surgery was collected and stained by H&E. The histological images in Fig. 9B–D, H–J revealed that a large amount of inflammatory cells were infiltrated into the MRSA- and *P. aeruginosa*-infected wounds in the Vaseline gauze, TPU and TPU/DA groups, which further confirmed the severe infection caused by bacteria, as observed in the macroscopic photographs (Figs. 7a, 8a). Nevertheless, such signs of infection were not observed in the subcutaneous tissues of wounds that were treated with TPU/NS2.5 membranes (Fig. 9E, K), and there were significantly less inflammatory cells in the TPU/NS2.5 group than in the Vaseline gauze, TPU and TPU/DA groups, according to the quantitative count results (Fig. 9F, L). These data further indicate that treatment with TPU/NS2.5 membranes can effectively prevent bacterial infections and maintain a natural microenvironment for the regeneration of cells and tissues.

**Fig. 10:**
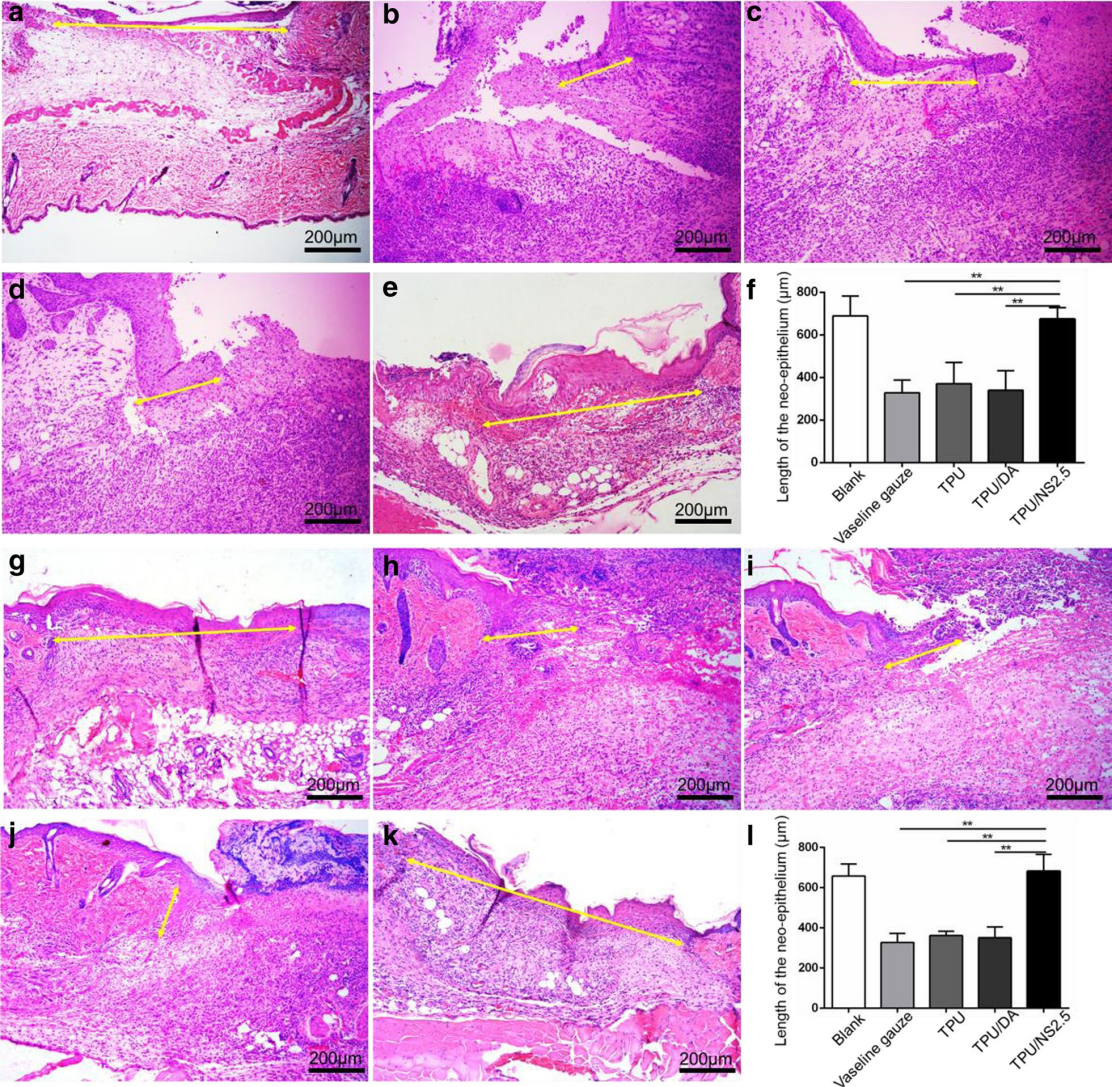
The effect of TPU/NS2.5 on re-epithelialization. Representative H&E staining images of **a**, **g** blank, **b**, **h** Vaseline gauze, **c**, **i** TPU, **d**, **j** TPU/DA and **e**, **k** TPU/NS2.5 groups at day 7 post-surgery. The images **a**–**e** indicate MRSA-infected wounds, and the images **g**–**k** indicate *P. aeruginosa*-infected wounds. The yellow double-headed arrows indicate the length of regenerated epidermis. Measurement of the length of regenerated epidermis of (**f**) MRSA-infected wounds and (**l**) *P. aeruginosa*-infected wounds. The values are shown as the mean ± SD (*n* = 5)

Restoration of the epidermal layers is a key factor in evaluating wound healing [54], therefore we investigated the effect of TPU/NS2.5 membranes on re-epithelialization based on H&E staining of sections. As shown in Fig. 10, the length of newly regenerated epidermis in the TPU/NS2.5 group was significantly longer than that in the Vaseline gauze, TPU and TPU/DA groups at day 7 post-surgery both for the MRSA- and *P. aeruginosa*-infected wounds, but was similar to that in the Blank group. This result indicated that compared with other treatments, the TPU/NS2.5 membrane was able to promote wound closure by accelerating the re-epithelialization process in the infected wounds, which may be due to its excellent antibacterial activity and good biocompatibility. More importantly, the application of TPU/NS2.5 membranes was also expected to provide a better quality of wound healing, as rapid re-epithelialization is known to reduce hypertrophic scar formation [55].

**Fig. 11:**
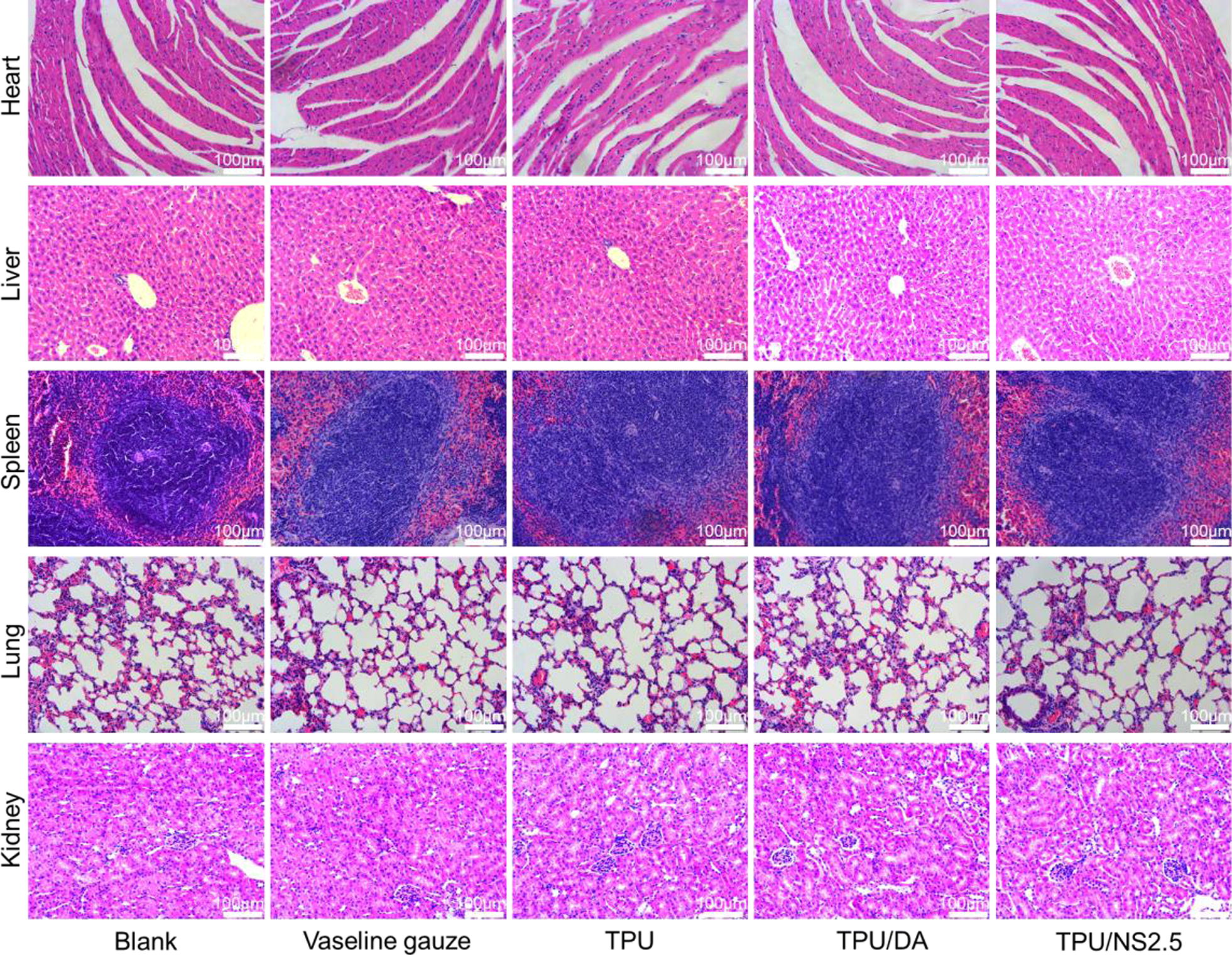
Evaluation of the in vivo toxicity of TPU/NS2.5 on major murine organs. Representative H&E staining images of heart, liver, spleen, lung and kidney at day 7 post-surgery

To further evaluate the in vivo biosafety of TPU/NS2.5 membranes, the major organs of mice in each group after 7 days of treatment were collected for histological analysis [36]. As shown in Fig. 11, similar to the Blank group, no obvious inflammatory lesions or appreciable abnormalities were observed in the heart, liver, spleen, lung and kidney of mice treated with TPU/NS2.5 membranes, which indicated that the prepared nanocomposite had acceptable biocompatibility in vivo.

Taken together, these results demonstrate that TPU/NS2.5 membranes can efficiently eliminate MRSA and *P. aeruginosa* infections in vivo, and maintain a non-septic and normal wound microenvironment for tissue regeneration, leading to rapid re-epithelialization and wound closure, while causing no measurable damage to normal tissues. Therefore, TPU/NS2.5 is a promising candidate for application in the management of infected wounds.

## Conclusions

In summary, we prepared a biocompatible, flexible and antibacterial wound dressing by incorporating nano-silver onto a porous TPU membrane using biomimetic polydopamine. The entire preparation process was facile, mild and eco-friendly. The TPU/NS2.5 dressing possessed strong mechanical strength and excellent flexibility, and exhibited acceptable antibacterial activity against *P. aeruginosa*, *E. coli*, *S. aureus* and MRSA while exhibiting no obvious toxicity to mammalian cells. Furthermore, by employing a bacteria-infected (MRSA or *P. aeruginosa*) wound model, we found the dressing could effectively prevent bacterial infection in vivo and promote wound healing by accelerating re-epithelialization. Therefore, the constructed TPU/NS2.5 membrane has great potential for biomedical applications such as wound management.
